# Isolation, characterization, and *in vitro* efficacy of phage Citro-6 against *Citrobacter freundii*-induced bovine mastitis

**DOI:** 10.3389/fmicb.2026.1879434

**Published:** 2026-07-03

**Authors:** Ziyi Wang, Hong Li, Rihua Xu, Ke Meng, Jun Zhu

**Affiliations:** 1 School of Life Sciences, Inner Mongolia University, Hohhot, Inner Mongolia, China; 2 Ejin Horo Animal Disease Prevention and Control Center, Ordos, Inner Mongolia, China; 3 School of Physical Science and Technology, Inner Mongolia University, Hohhot, Inner Mongolia, China

**Keywords:** BMECs, bovine mastitis, *Citrobacter freundii*, *in vitro* efficacy, phage therapy

## Abstract

**Introduction:**

Bovine mastitis is a prevalent infectious disease in dairy herds worldwide, resulting in substantial economic losses. *Citrobacter freundii* has emerged as an opportunistic pathogen associated with mastitis, and its increasing antibiotic resistance poses significant therapeutic challenges.

**Methods:**

In this study, a novel lytic phage, Citro-6, was isolated from dairy farm sewage and fecal samples using *C. freundii* Z6 as the host bacterium. The phage was characterized through morphological and genomic analyses. Its host range, stability under varying temperature and pH conditions, and effects on bacterial adhesion, invasion, cytotoxicity, and pro-inflammatory cytokine expression in bovine mammary epithelial cells were evaluated in vitro.

**Results:**

Morphological and genomic analyses identified Citro-6 as a member of the class Caudoviricetes. It encodes a complete endolysin sequence with an intact catalytic domain and lacks virulence or antibiotic resistance genes. Citro-6 exhibited broad lytic activity, lysing 83.3% of tested *Citrobacter* strains, and remained stable at temperatures up to 45 °C and across a pH range of 5.0 to 9.0. In cellular assays, Citro-6 treatment significantly reduced bacterial adhesion and invasion by approximately 1.66 and 1.19 log units, respectively. Furthermore, it effectively attenuated pathogen-induced cytotoxicity, reducing cell death by approximately 75.2% at 8 h post-infection compared with the Z6-infected group. Citro-6 also prevented morphological cellular damage and suppressed the overexpression of pro-inflammatory cytokines, resulting in decreases in IL-1β and TNF-*α* concentrations of approximately 37.1% and 47.6%, respectively, relative to the infected control.

**Discussion:**

These findings demonstrate that phage Citro-6 is a promising biocontrol candidate against *C. freundii*-induced bovine mastitis; however, further *in vivo* evaluation is warranted to confirm its therapeutic potential.

## 1 Introduction

Bovine mastitis remains one of the most economically detrimental diseases affecting the dairy industry worldwide. It leads to substantial financial losses through reduced milk yield, altered milk composition, increased culling rates, and elevated treatment and labor costs (Ruegg, 2017). Beyond its direct economic impact, mastitis also compromises animal welfare and represents a public health concern due to antimicrobial use in dairy cattle and the potential transmission of pathogenic microorganisms through the food chain (Soyoun et al., 2022). Global annual losses associated with mastitis were estimated in 2016 to range from €19.7 to €32 billion, underscoring the urgent need for effective control strategies (Nale and McEwan, 2023).

Although mastitis can be caused by over 140 different microorganisms, bacterial pathogens remain the predominant etiological agents (Sharun et al., 2021). Among Gram-negative bacteria associated with environmental mastitis, *C. freundii* has emerged as an opportunistic pathogen of increasing concern (Schukken et al., 2012). This coliform species, commonly found in the environment of dairy farms including bedding, manure, and contaminated water sources, can cause both clinical and subclinical intramammary infections (Chotigarpa et al., 2019). Furthermore, *C. freundii* and related Enterobacteriaceae have developed adaptive survival mechanisms in dairy environments, such as forming robust biofilms on mammary epithelium and developing resistance to common disinfectants. These adaptations prolong the infection cycle and increase recurrence rates (Pedersen et al., 2021).

Antibiotic therapy, primarily administered via intramammary infusion, remains the mainstay for treating bovine mastitis (Yu et al., 2025). However, its efficacy is increasingly compromised by (i) the emergence of antimicrobial resistance among Enterobacteriaceae due to widespread antibiotic use (Jiang et al., 2024; Saidani et al., 2018), with *C. freundii*, an environmental mastitis pathogen, showing concerning resistance profiles: the single isolate tested in one study was resistant to ampicillin (AMP) (Abdel-Hameed et al., 2025); another study reported that all three *C. freundii* isolates were resistant to ceftazidime, cefuroxime, cefixime, and augmentin (Aromolaran et al., 2024); and a long-term surveillance study found an overall antimicrobial resistance rate of 40.8% for this pathogen across multiple agents (Jessberger et al., 2026); (ii) intrinsic limitations of intramammary administration including poor tissue penetration and rapid drug elimination (Ban-Cucerzan et al., 2026), and (iii) milk residue concerns necessitating costly withdrawal periods (Maalaoui et al., 2025). These limitations underscore the urgent need for alternative therapeutic strategies.

Bacteriophages (phages), viruses that specifically infect and lyse bacteria, have garnered renewed interest as promising alternatives or adjuncts to antibiotic therapy (Debruyn et al., 2025). Phages offer several inherent advantages, including high host specificity, self-amplification at the site of infection, minimal disruption of commensal microbiota, and the ability to penetrate biofilms and target intracellular bacteria (Ash et al., 2025). Unlike broad-spectrum antibiotics, phages can be selected to target specific pathogens, thereby reducing off-target effects and preserving the beneficial microbial ecosystem (Mohammadi et al., 2025). Moreover, the continuous co-evolution of phages with their bacterial hosts enables them to overcome bacterial resistance mechanisms, and phage cocktails can be formulated to minimize the emergence of phage-resistant mutants (Li et al., 2024a). Previous studies have demonstrated the potential of phage therapy against various mastitis-associated pathogens, including *Staphylococcus aureus*, Enterobacteriaceae such as *Escherichia coli*, and *Klebsiella pneumoniae*, both *in vitro* and *in vivo* (Banar et al., 2025; Duc et al., 2024; Liang et al., 2023). Phage cocktails have been shown to significantly reduce bacterial loads in bovine mammary epithelial cells (bMECs) and in murine mastitis models, while also attenuating inflammatory responses (Shi et al., 2021). However, despite growing evidence supporting phage therapy for mastitis caused by Gram-positive and some Gram-negative pathogens, limited information is available regarding the application of phages specifically targeting *C. freundii* associated with bovine mastitis.

To date, only a few lytic phages targeting *C. freundii* have been reported, including YZU-L1 (Tang et al., 2023), IME-JL8 (Jia et al., 2020), and K1M (Xie et al., 2025). However, these studies focused on applications such as aquaculture or general environmental disinfection, and none were developed for the treatment of bovine mastitis. To our knowledge, no phage has yet been reported specifically targeting *C. freundii* isolates of bovine mastitis origin or evaluated in a mastitis-relevant cell model.

The objective of this study was to isolate and characterize a novel lytic phage (Citro-6) targeting *C. freundii* Z6 from clinical mastitis milk. The novelty is threefold: (i) the phage was isolated from dairy farm sewage, while its host is of mastitis origin; (ii) we systematically assessed its biological, genomic, host range, and stability profiles; and (iii) we evaluated its protective effects on bMECs against *C. freundii* infection, including bacterial adhesion, invasion, cell morphology preservation, and anti-inflammatory cytokine modulation.

## 2 Materials and methods

### 2.1 Bacterial strains and culture conditions

In this study, *C. freundii* Z6 was isolated from milk samples obtained from cows with clinical mastitis using sheep blood agar for purification, and was identified by Gram staining, biochemical identification tubes, and 16S rRNA sequencing. The 16S rRNA gene sequence of *C. freundii* Z6 has been deposited in the National Microbiology Data Center (NMDC) with accession number NMDCN000D0QR^1^. For subsequent experiments, the strain was cultured in LB liquid medium at 37 °C with shaking at 180 rpm until it reached the logarithmic growth phase.

### 2.2 Antimicrobial susceptibility testing and MAR index

Antimicrobial susceptibility testing (AST) was performed only for the Z6 strain because it was the primary clinical isolate used as the target host for phage isolation and subsequent therapeutic evaluation. The other 11 strains employed in the host range determination were not subjected to AST, as they were included solely for host range assessment and not as therapeutic targets. The antimicrobial resistance profile of the isolate was assessed using a panel of 10 conventional antibiotics (Changde Bikeman Biotechnology Co., Ltd.), including penicillin (PEN), ceftriaxone (CTR), AMP, gentamicin (GEN), tetracycline (TET), chloramphenicol (C), trimethoprim-sulfamethoxazole (SXT), lincomycin (MY), ciprofloxacin (CIP), and erythromycin (E) (concentrations as per CLSI M100-S32). The panel includes antibiotics active against both Gram-positive and Gram-negative bacteria because bovine mastitis is often a mixed infection involving multiple bacterial genera, and antibiotics used on farms are not restricted to Gram-negative-specific agents. This selection reflects real-world clinical practice and allows a comprehensive assessment of the Z6 isolate’s resistance profile.

After performing AST, the multiple antibiotic resistance (MAR) index for *C. freundii* Z6 was calculated using the following formula.


$$\mathrm{MAR}\;\text{index}=\frac{\begin{array}{l} \text{Number of antibiotics to which the}\;\\ \text{isolate was resistant} \end{array}}{\text{Total number of antibiotics tested}}$$

### 2.3 Bacteriophage isolation and propagation

Sewage and cow fecal samples were collected from a dairy farm in Inner Mongolia, China. Sample treatment was performed with slight modifications based on a previously described method (Li et al., 2024b). Using *C. freundii* Z6 as the host bacterium, the phage was isolated and purified by the spot test and double-layer agar method (Cho et al., 2025). A single purified plaque was eluted in 500 μL of SM buffer. The eluate (100 μL) was mixed with 100 μL of host bacterial suspension and allowed to adsorb, after which the mixture was added to 10 mL of a logarithmic-phase host bacterial culture. The culture was incubated at 37 °C with shaking at 180 rpm for 4–6 h until clearing of the culture was observed. The lysate was centrifuged at 8000 rpm for 10 min, and the supernatant was filtered through a 0.22 μm membrane to obtain a high-titer phage lysate. The titer of the phage suspension was subsequently determined using the double-layer agar method (Göller et al., 2021).

### 2.4 Morphological analysis by transmission electron microscopy

Phage morphology was examined using phosphotungstic acid negative staining. A drop of high-titer phage lysate was placed on a carbon-coated copper grid and allowed to adsorb for 5 min. Excess liquid was removed by blotting with filter paper. The grid was then negatively stained with 2% phosphotungstic acid (PTA, pH 7.0) for 10 min. After air-drying, the phage particles were observed under a Hitachi 7,800 transmission electron microscope (TEM) at an accelerating voltage of 80 kV.

### 2.5 Host range relative efficiency of plating

A panel of 12 *Citrobacter* strains was used to determine the host range of the phage Citro-6. This panel included one strain each of *Citrobacter rodentium*, *C. amalonaticus*, *C. braakii*, *C. youngae*, *C. sedlakii*, and *Citrobacter* sp. DH3, as well as two strains of *C. koseri* and four strains of *C. freundii*. Among these, two strains of *C. freundii* and one strain of *C. sedlakii* were isolated from clinical mastitis milk samples, whereas the remaining strains were provided by Tianjin Jinkebio Technology Co., Ltd. (Tianjin, China). Susceptibility testing was performed using the spot-on-plate method and double-layer agar method as previously described, and for sensitive strains, the efficiency of plating (EOP) was further determined (Gorodnichev et al., 2025). Plating with the known host bacterium served as a positive control, whereas plating with non-susceptible strains served as a negative control.


$$\mathrm{EOP}=\frac{\begin{array}{l} \text{Average number of plaque}-\\ \text{forming units}\;(\mathrm{PFU})\;\mathrm{on}\;\text{the test strain} \end{array}}{\begin{array}{l} \text{Average number of plaque}-\text{forming units}\;(\mathrm{PFU})\;\\ \mathrm{on}\;\text{the reference strain}\;(\text{host strain}) \end{array}}$$

### 2.6 One-step growth curve

The method for determining the optimal multiplicity of infection (MOI) is slightly modified from the one described earlier (Han et al., 2022). Based on the optimal MOI value, a one-step growth curve was plotted using a slightly modified version of the method developed by Kuźmińska-Bajor et al. (2021) to determine the phage’s incubation period, growth phase, and burst size. Briefly, the phage was added to a logarithmic-phase host bacterial culture at the optimal MOI and incubated at 37 °C with shaking at 180 rpm. Samples were collected at regular intervals, and phage titers were immediately determined using the double-layer agar method.

### 2.7 Thermal and pH stability

To assess phage Citro-6 stability under varying environmental conditions, phages were incubated for 1 h at different temperatures (35–75 °C) and pH values (3–11) according to previously described methods (Guo et al., 2021). Phage titers were immediately determined using the double-layer agar method.

### 2.8 Genome sequencing and bioinformatics analysis

Nucleic acid extraction from phage Citro-6 was performed using the TaKaRa MiniBEST Viral RNA/DNA Extraction Kit. Following extraction, nucleic acid hydrolase was employed to preliminarily identify the genetic type as double-stranded DNA via agarose gel electrophoresis. Quality assessment was conducted using Qubit fluorescence quantification and Nanodrop spectrophotometry to ensure integrity, concentration, and purity (A260/A280 and A260/A230 ratios) met standards, enabling subsequent library preparation and sequencing. Qualified constructed libraries underwent pooling and sequencing. Nucleic acid extraction and whole-genome sequencing of the phage were performed by Guangdong Meige Gene Technology Co., Ltd.

After sequencing, genome annotation and visualization were performed using the online server PHASTEST^2^, which predicts open reading frames (ORFs) and assigns putative gene functions. Based on the prediction results, endolysins and holins within the lysis module, which are involved in host lysis, were selected for further amino acid sequence analysis. Conserved domains in these candidate proteins were identified using the NCBI CD-Search tool to confirm their functions. Subsequently, Prokka (v1.14.6) was used to refine and supplement the annotation of protein-coding sequences across the whole genome. To assess the safety of the phage, the predicted proteome was submitted to the Comprehensive Antibiotic Resistance Database (CARD)^3^, and potential antibiotic resistance genes were identified using the Resistance Gene Identifier (RGI) software integrated within the platform. Homology searches of the whole-genome sequences were conducted using the NCBI BLAST algorithm^4^. A phylogenetic tree was constructed based on the complete genome sequences of the phage and its closely related relatives via the neighbor-joining algorithm implemented in MEGA11 to clarify the taxonomic classification and evolutionary relationships of the phage, with evolutionary distances calculated using the p-distance model and bootstrap analysis set at 1,000 replicates for reliability assessment.

### 2.9 Antibacterial efficacy in UHT whole milk

Following the method of Cunha et al. (2025) with minor modifications, *C. freundii* Z6 and phage Citro-6 cultured to the logarithmic growth phase were mixed at a 1:1 ratio with an optimal MOI (MOI = 0.01) and inoculated into 5 mL of ultra-high-temperature instantaneous sterilization (UHT) whole milk to form the experimental group. The positive control group was inoculated with *C. freundii* Z6 only, substituting sterile PBS buffer for phage inoculation. The negative control used only sterile PBS buffer instead of both host bacteria and phage. All groups were incubated at 37 °C, with aliquots collected at 0, 3, 6, 12, 24, and 48 h. All samples underwent serial dilutions. Bacterial concentration (CFU/mL) was determined using the spread plate method, while phage titer (PFU/mL) was measured via the double-layer agar method.

### 2.10 Bovine mammary epithelial cell culture and treatment

The bMECs were obtained from Shanghai Fuheng Biotechnology Co., Ltd. (Shanghai, China). Because *C. freundii* is an emerging pathogen associated with bovine mastitis, and bovine mammary epithelial cells represent the primary target of bacterial infection in the udder, we used bMECs as a physiologically relevant *in vitro* model to evaluate bacterial adhesion, invasion, and phage-mediated protection. The cells were cultured in Dulbecco’s modified Eagle’s medium (DMEM) supplemented with 10% (v/v) fetal bovine serum (FBS) at 37 °C in a humidified atmosphere containing 5% CO₂. Cells from passage 5 were used for all experiments to ensure consistency. The bMECs were treated with or without *C. freundii* Z6 (MOI, ratio of bacteria to bMECs = 10:1) (Shi et al., 2021) and phage Citro-6 (MOI, ratio of phage to *C. freundii* Z6 = 0.01:1), resulting in the following experimental groups: control (untreated cells); *C. freundii* Z6 infection group (*C. freundii* Z6 alone); and *C. freundii* Z6 plus phage Citro-6 group (*C. freundii* Z6 + phage Citro-6).

### 2.11 Bacterial adhesion and invasion assay

Adhesion and invasion assays were performed using the previously described method with minor modifications (Shi et al., 2021). The bMECs were seeded in 6-well plates and cultured until reaching approximately 80% confluence. Cells were washed with phosphate-buffered saline (PBS), and the medium was replaced with DMEM supplemented with 1% (v/v) FBS. The bMECs were then infected with *C. freundii* Z6 at a MOI (ratio of bacteria to cells) of 10:1 and incubated at 37 °C in a humidified atmosphere containing 5% CO₂ for 0.5 h to allow bacterial adhesion. Subsequently, phage Citro-6 was added at the optimal MOI of 0.01 (ratio of phage to bacteria), and cells were further incubated for 0.5, 1, 2, 4, 6, and 8 h.

To assess bacterial adhesion, cells were washed with PBS to remove non-adherent bacteria after each time point, and the cell suspensions were collected, serially diluted 10-fold, and plated on LB agar for colony counting.

For invasion assays, after incubation, cells were washed with PBS and treated with 1 mL of DMEM containing kanamycin (100 μg/mL) for 1 h at 37 °C to kill extracellular bacteria. The culture medium was then removed, and cells were lysed with 0.5% Triton X-100. The lysates were serially diluted 10-fold and plated on LB agar as described above. Colonies were counted, and the invasion rate of *C. freundii* Z6 into bMECs was calculated.

### 2.12 Lactate dehydrogenase (LDH) release assay

The bMECs were seeded in 96-well plates at a density of 1 × 10^5^ cells per well and cultured until reaching approximately 80% confluence. The medium was then replaced with DMEM supplemented with 1% (v/v) FBS, and cells were treated with *C. freundii* Z6 and phage Citro-6 as described above. After incubation for 1, 2, 4, 6, or 8 h, cell culture supernatants were collected and centrifuged at 1,000 × g for 5 min at 4 °C. Lactate dehydrogenase (LDH) release was measured using an LDH assay kit (Wuhan Servicebio Technology Co., Ltd., Wuhan, China) according to the manufacturer’s instructions. Absorbance was measured at 490 nm using a microplate reader, with 630 nm serving as the reference wavelength for dual-wavelength measurement.

### 2.13 Morphological observation of bMECs

The bMECs were seeded in 6-well plates containing glass coverslips and cultured until reaching approximately 80% confluence. Cells were then treated with *C. freundii* Z6 and phage Citro-6 as described above, and incubated at 37 °C in a humidified atmosphere containing 5% CO₂ for 2, 4, 6, or 8 h. After treatment, cells were collected, fixed in 4% paraformaldehyde for 15 min, washed three times with PBS. Modified hematoxylin–eosin (HE) staining was performed using a commercial kit (Beijing Solarbio Science & Technology Co., Ltd., Beijing, China) according to the manufacturer’s instructions. Cells were then dehydrated through an ascending alcohol gradient (75, 85, 95, and 100%) and mounted with neutral balsam. Morphological changes were observed under a light microscope at 40 × magnification.

### 2.14 Cytokine concentration detection

Cell culture supernatants were collected and centrifuged at 1,000 × g for 20 min at 4 °C to remove debris. The concentrations of inflammatory cytokines (e.g., TNF-*α* and IL-1β) in the clarified supernatants were measured using commercial ELISA kits (Shanghai Jianglai Industrial Co., Ltd., Shanghai, China) according to the manufacturer’s instructions.

### 2.15 Statistical analysis

All statistical analyses were performed using Origin 2021 (OriginLab Corporation, Northampton, MA) and SPSS 27.0.1 (IBM Corp., Armonk, NY). Comparisons among multiple groups were conducted using one-way analysis of variance (ANOVA) to assess differences between groups. Post-hoc pairwise comparisons were performed using Duncan’s multiple range test. Most assays were performed in triplicate, and data were expressed as the mean ± standard deviation (SD) of three independent experiments. Differences were considered statistically significant at *p < 0.05 and extremely significant at **p < 0.01.

## 3 Results

### 3.1 Isolation and identification of *C. freundii* and phage Citro-6

Antimicrobial susceptibility of *C. freundii* Z6 was determined by the disk diffusion method and interpreted per Clinical and Laboratory Standards Institute (CLSI) guidelines (CLSI, 2022). The isolate was resistant to PEN, AMP, MY, and E. Among these, resistance to PEN, MY, and E is intrinsic to *C. freundii* due to outer membrane impermeability and is not clinically meaningful for this Gram-negative isolate. In contrast, resistance to AMP represents an acquired, clinically significant trait. Overall, the isolate was resistant to 4 of the 10 antibiotics tested, yielding a MAR index of 0.4.

**Figure 1:**
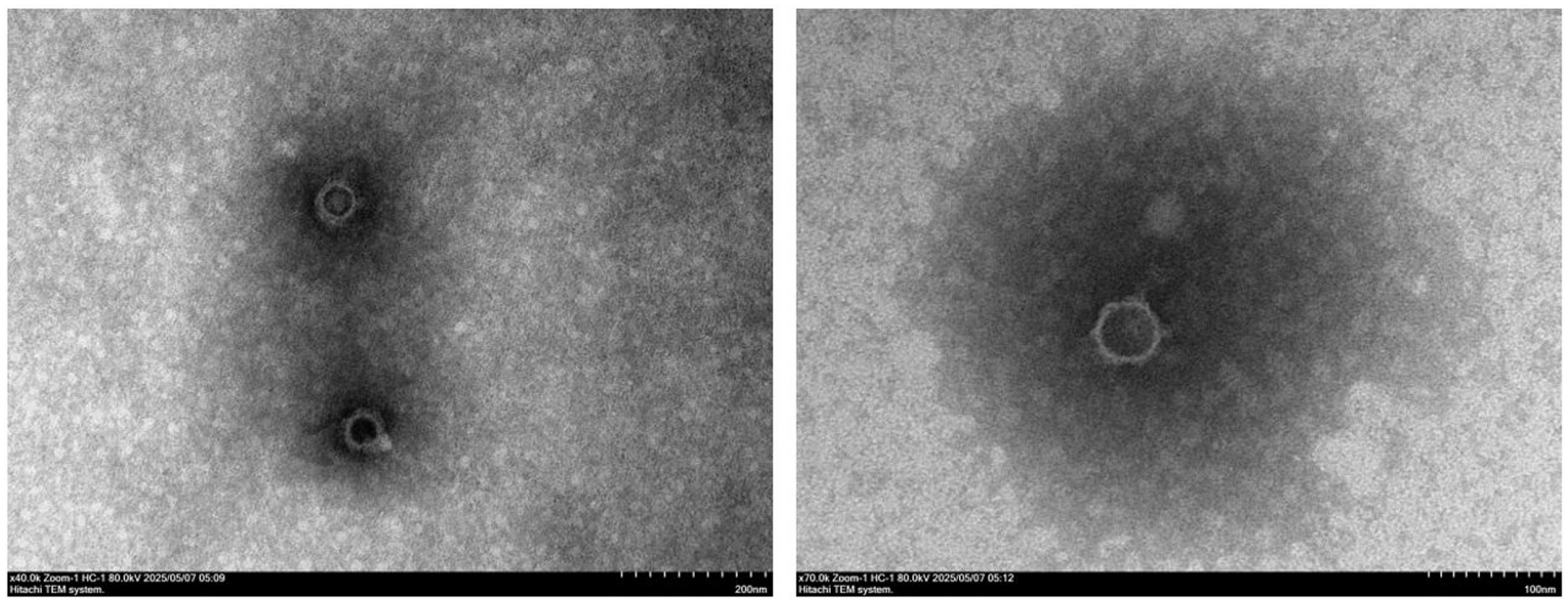
Transmission electron microscope images of Citro-6. Magnifications are 40,000 × and 70,000×, with scale bars of 200 nm and 100 nm, respectively.

Transmission electron microscopy (TEM) revealed that phage Citro-6 possesses an icosahedral head approximately 54 nm in diameter and a long, slender, non-contractile tail approximately 90 nm in length. Based on its morphological characteristics, and according to the 2022 update of the ICTV bacterial viruses subcommittee (Turner et al., 2023), Citro-6 belongs to the class Caudoviricetes and exhibits typical siphovirus morphology (Figure 1).

### 3.2 Host range and lytic activity

Phage Citro-6 exhibits lytic activity not only against its host bacterium *C. freundii* Z6 but also against most tested strains (83.3%), demonstrating a broad intrageneric lytic spectrum. However, its lytic capacity varies among different strains, as shown in Table 1.

**Table 1:** Lysis spectrum of bacteriophage Citro-6.

Test strains	Spot test^a^	Average titer (PFU/mL)^b^	EOP^c^
*Citrobacter rodentium*	+	4.4 × 10^9^	0.506
*Citrobacter koseri* 1	+	9.5 × 10^9^	1.09
*Citrobacter amalonaticus*	+	3.6 × 10^9^	0.414
*Citrobacter braakii*	+	5.7 × 10^9^	0.655
*Citrobacter koseri* 2	+	1.3 × 10^9^	0.149
*Citrobacter youngae*	+	8.9 × 10^9^	1.02
*Citrobacter freundii* Z6	+	8.7 × 10^9^	1
*Citrobacter freundii* Z21	−	−	−
*Citrobacter freundii* 1	+	1.1 × 10^10^	1.26
*Citrobacter freundii* 2	+	1.5 × 10^9^	0.172
*Citrobacter* DH3	+	1.5 × 10^10^	1.72
*Citrobacter sedlakii* W-6	−	−	−

^a^Spot test results: “+” indicates visible lysis; “–” indicates no lysis. ^b^Values are the average titers from three independent experiments (each performed in triplicate). ^c^EOP was calculated as the ratio of the phage titer (PFU/mL) on the test strain to that on the permissive host *C. freundii* Z6 (the reference strain, EOP = 1.0).

### 3.3 Growth kinetics and stability

**Figure 2:**
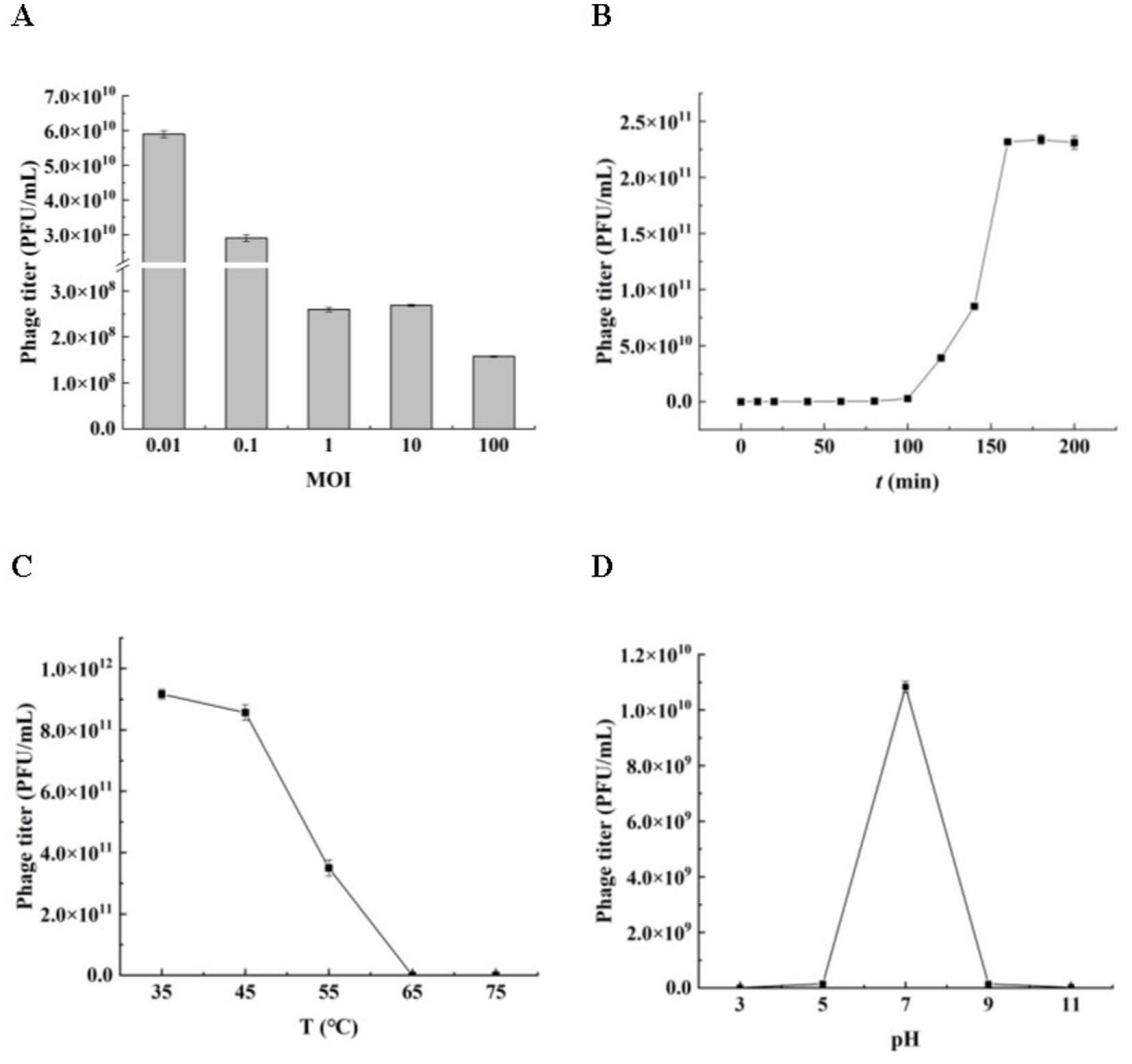
Biological characteristics of bacteriophage Citro-6. **(A)** Optimal multiplicity of infection for bacteriophage Citro-6. **(B)** One-step growth curve of phage Citro-6 at MOI 0.01. The burst size was calculated as the final phage titer divided by the initial phage titer after the latent period, yielding approximately 1.5 × 10^7^ PFU/cell. **(C)** Thermal stability of bacteriophage Citro-6. **(D)** pH stability of phage Citro-6. Actual titers: pH 5 = 1.5 × 10^8^ PFU/mL; pH 9 = 1.4 × 10^8^ PFU/mL. Although the pH 5 and pH 9 bar appears near baseline due to low titer at pH 3, the phage retains substantial activity at pH 5 and pH 9.

MOI experiments revealed that phage Citro-6 exhibited the highest titer at an MOI of 0.01, reaching approximately 5.9 × 10^10^ PFU/mL (Figure 2A). A one-step growth curve assay conducted at the optimal MOI of 0.01 revealed that phage Citro-6 exhibited a latency period of 100 min, a lysis phase between 100 and 160 min, and a substantial burst index, with a burst size of approximately 1.5 × 10^7^ PFU/cell (Figure 2B).

The titer of phage Citro-6 remained relatively stable at temperatures up to 45 °C but decreased significantly when exposed to temperatures above 55 °C. At 65 °C and above, phage infectivity was completely lost (Figure 2C). Regarding pH stability, incubation for 1 h under strong acidic (pH ≤ 4.0) or strong alkaline (pH ≥ 10.0) conditions resulted in a significant reduction in phage titer by approximately 4.0 log PFU/mL compared to the optimal pH (pH = 7.0) control (Figure 2D).

### 3.4 Genomic features

**Figure 3:**
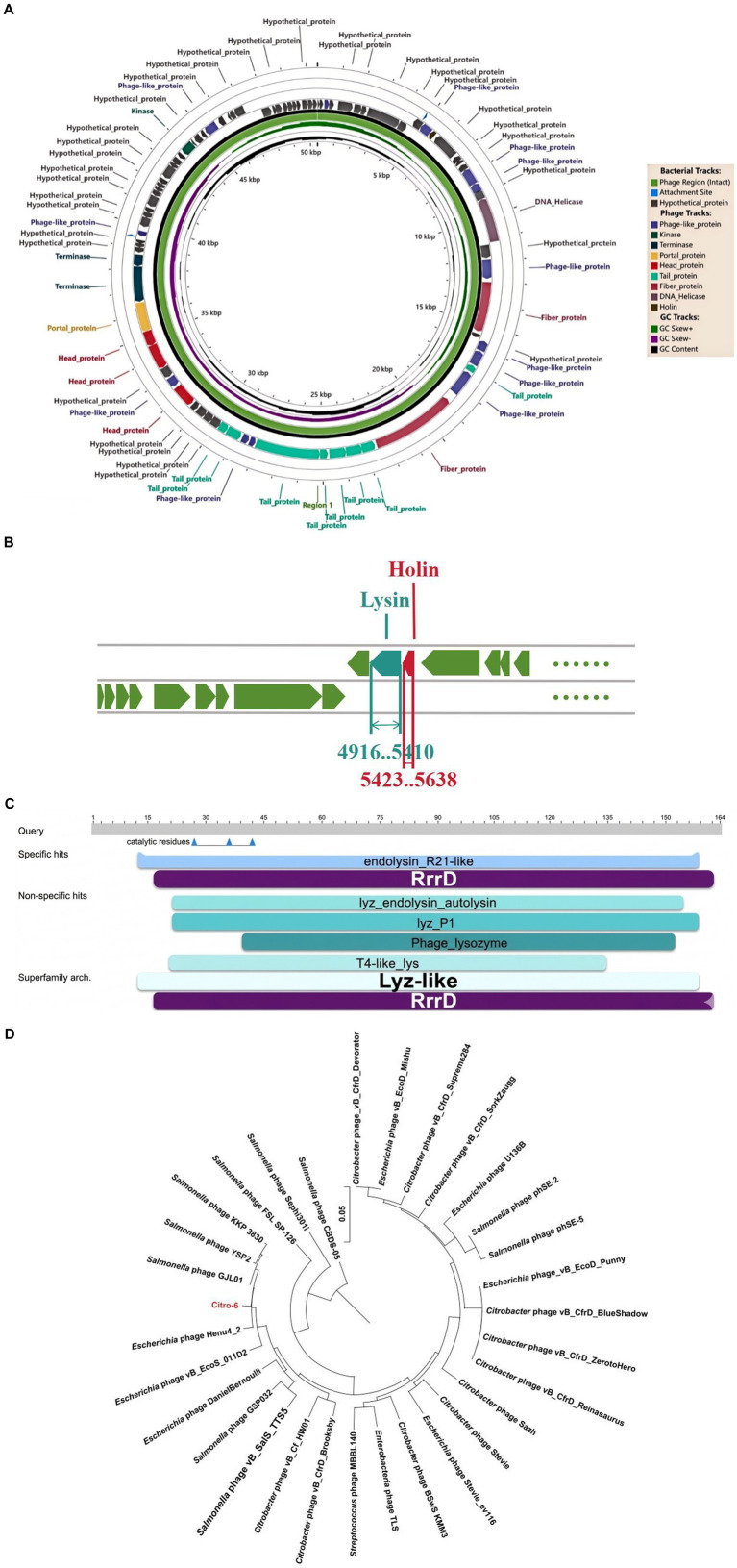
Genomic information on bacteriophage Citro-6. **(A)** Complete genome map of phage Citro-6. The whole-genome circle diagram is annotated with gene annotation results in distinct colors, corresponding to functions in the legend. **(B)** Lysis functional modules of phage Citro-6. **(C)** Predicted conserved domains of lysin. **(D)** Phylogenetic tree of the phage Citro-6 genome.

Whole-genome sequencing analysis of the phage Citro-6 reveals a double-stranded circular DNA genome spanning 50,436 bp with a G + C content of 43.31%. The genomic circular diagram is shown in Figure 3A. A total of 87 ORFs were predicted, comprising 85 phage-derived genes. Additionally, two bacterial-derived genetic elements were identified by PHASTEST as the attachment site sequences *attL* and *attR*, which are typically involved in site-specific recombination for phage integration. Phage-specific genes accounted for 97.70% of the genome, indicating a typical virulent phage genome with extremely low integration of host-derived genes. Complete structural and functional gene clusters were identified, including head proteins, portal proteins, tail proteins, and filament proteins involved in structural assembly. Additionally, functional genes related to phage nucleic acid metabolism, packaging, and lysis (e.g., endonuclease, DNA helicase, kinase, and holin) were annotated, collectively constituting the complete phage replication and infection machinery. The genome contains one complete phage region with no apparent fragmentation of phage genes. Gene clusters exhibit continuous, conserved modular arrangements consistent with the characteristics of a typical tailed phage genome. Numerous putative protein-coding sequences remain uncharacterized as phage-specific genes. Concurrently, multiple phage-like protein homologs indicate sequence conservation and evolutionary relationships with known phage families. No antibiotic resistance genes or pathogenic virulence factors were detected. Based on the genomic structure, functional gene composition, and key genetic element characteristics of phage Citro-6, it possesses a complete lytic replication pathway and lacks the ability to undergo a lysogenic cycle, classifying it as a lytic phage.

A typical lysis module has been identified in the genome of bacteriophage Citro-6. This module consists of the lysin-coding gene (4916.0.5410) and the holin-coding gene (5423.0.5638), which are arranged adjacent to each other with a 13 bp spacer; both are located on the negative strand, as shown in Figure 3B. The lysin gene is 495 bp in length and encodes 164 amino acids, the full sequence of which is provided in Supplementary file S1. Predicted analysis of conserved domains indicates that this endolysin contains a typical endolysin_R21-like domain, belonging to the Lyz-like lysozyme superfamily, and also matches the RrrD domain (Figure 3C). The sequence contains well-defined catalytic residue sites, suggesting that it belongs to the glycosidase-type phage endolysin class. It exerts lytic function by hydrolyzing bacterial cell wall peptidoglycan and serves as a key functional protein for the phage to release its progeny particles. The perforin gene is 216 bp in length and encodes 71 amino acids. Its amino acid sequence is also provided in Supplementary file S1. It forms pores in the host cell membrane, assisting the endolysin in penetrating the cell membrane barrier. Together, these two proteins coordinate the timing of phage lysis of the host, ensuring the lysis process proceeds in an orderly manner. From a comparative genomics perspective, the endolysin and perforin genes of this bacteriophage show high homology with those of *Salmonella* bacteriophage YSP2 (e-values of 3.0e-118 and 2.92e-44, respectively), indicating that this lysis system is relatively conserved among Enterobacteriaceae bacteriophages. *C. freundii* and *Salmonella* belong to the same family, Enterobacteriaceae, and their cell wall peptidoglycan structures are similar; this may be the structural basis for the functional conservation of lysis genes across genera. The endolysin sequence is intact and contains a complete catalytic domain, suggesting that it can independently perform lytic functions. The identification of this lytic system provides a molecular basis for further investigation of the phage’s lytic mechanism and its potential application as an antimicrobial agent.

To further evaluate the safety of the phage therapy candidate strain Citro-6, its antibiotic resistance genes and pathogenic virulence factors were annotated. The results showed that no genes associated with antibiotic resistance or virulence factors associated with pathogenic bacteria were detected in the genome of phage Citro-6. This indicates that the phage does not pose a potential risk of transmitting antibiotic resistance or virulence factors through horizontal gene transfer, and that its genetic background is safe, making it suitable for further development in antimicrobial applications.

To clarify the phylogenetic position and evolutionary relationships of phage Citro-6, a whole-genome phylogenetic tree was constructed using the Neighbor-Joining (NJ) method, and homology sequence alignment analysis was performed. The results are shown in Figure 3D. Phage Citro-6 did not cluster with phages of the genus *Citrobacter*, but instead formed a distinct evolutionary clade with extremely close genetic proximity to *Salmonella* phages such as GJL01 and YSP2. Furthermore, whole-genome sequence alignment results confirmed that Citro-6 shares 98.521% sequence identity with the reference sequence KY657202.1 (*Salmonella* phage YSP2 (Deng et al., 2023)), with an effective alignment length of 30,703 bp, indicating extremely high overall sequence homology between the two. This result not only aligns closely with the phylogenetic tree topology but also clearly reveals that Citro-6 belongs to the *Salmonella* phage group rather than the *Citrobacter* phage group in terms of evolutionary classification, suggesting that it may have acquired the ability to infect *C. freundii* through horizontal gene transfer, while maintaining high conservation in genomic composition and functional modules with the *Salmonella* phage YSP2. Combined with taxonomic annotation data, phage Citro-6 was classified as belonging to the genus *Tlsvirus* within the class Caudoviricetes. It represents a novel lytic phage within this genus characterized by highly conserved sequences and a recent co-evolutionary origin with known homologous phages. The whole-genome sequence data were deposited in National Microbiology Data Center^5^ with accession number NMDC60315064.

### 3.5 Efficacy in milk

**Figure 4:**
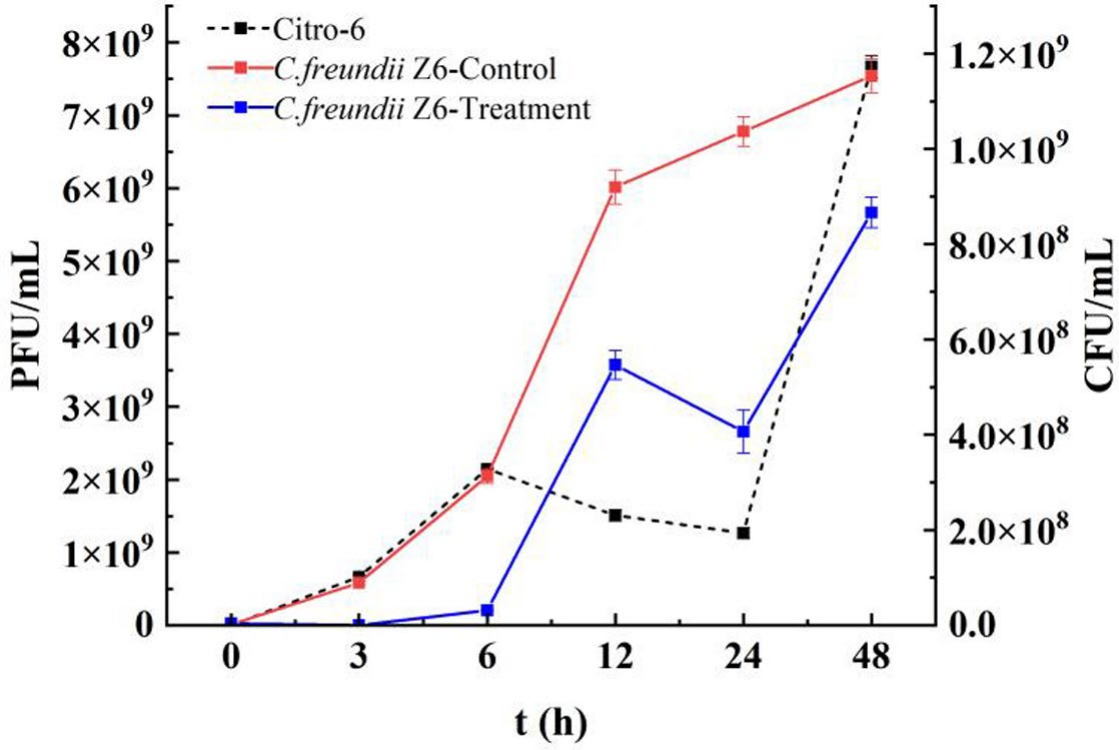
Bactericidal activity of phage Citro-6 against *C. freundii* Z6 in UHT whole-fat milk over 48 h. The graph illustrates the bacterial load (*C. freundii* Z6 CFU/mL) in untreated samples (red line, “*C. freundii* Z6-Control”) and the reduction in bacterial counts following phage treatment (blue line, “*C. freundii* Z6-Treatment”). Phage replication is represented by titers in PFU/mL (dotted black line, “Citro-6”).

In UHT whole milk, only the control group inoculated with *C. freundii* Z6 exhibited rapid growth throughout the entire incubation period, reaching 1.15 × 10^9^ CFU/mL by 48 h. In contrast, the phage Citro-6-treated group significantly suppressed Z6 growth during the initial incubation phase (0–6 h), with bacterial counts reduced by 2–3 orders of magnitude compared to the control group (*p* < 0.05). A brief proliferation occurred between 6–12 h, but the overall bacterial count remained lower than the control group within 24 h. At 48 h, the final bacterial count in the phage-treated group was 8.7 × 10^8^ CFU/mL, lower than that of the control group at the same time point (Figure 4). This indicates that phage Citro-6 maintains relatively stable lytic activity even in complex environments such as dairy matrices.

### 3.6 Protection of bMECs

**Figure 5:**
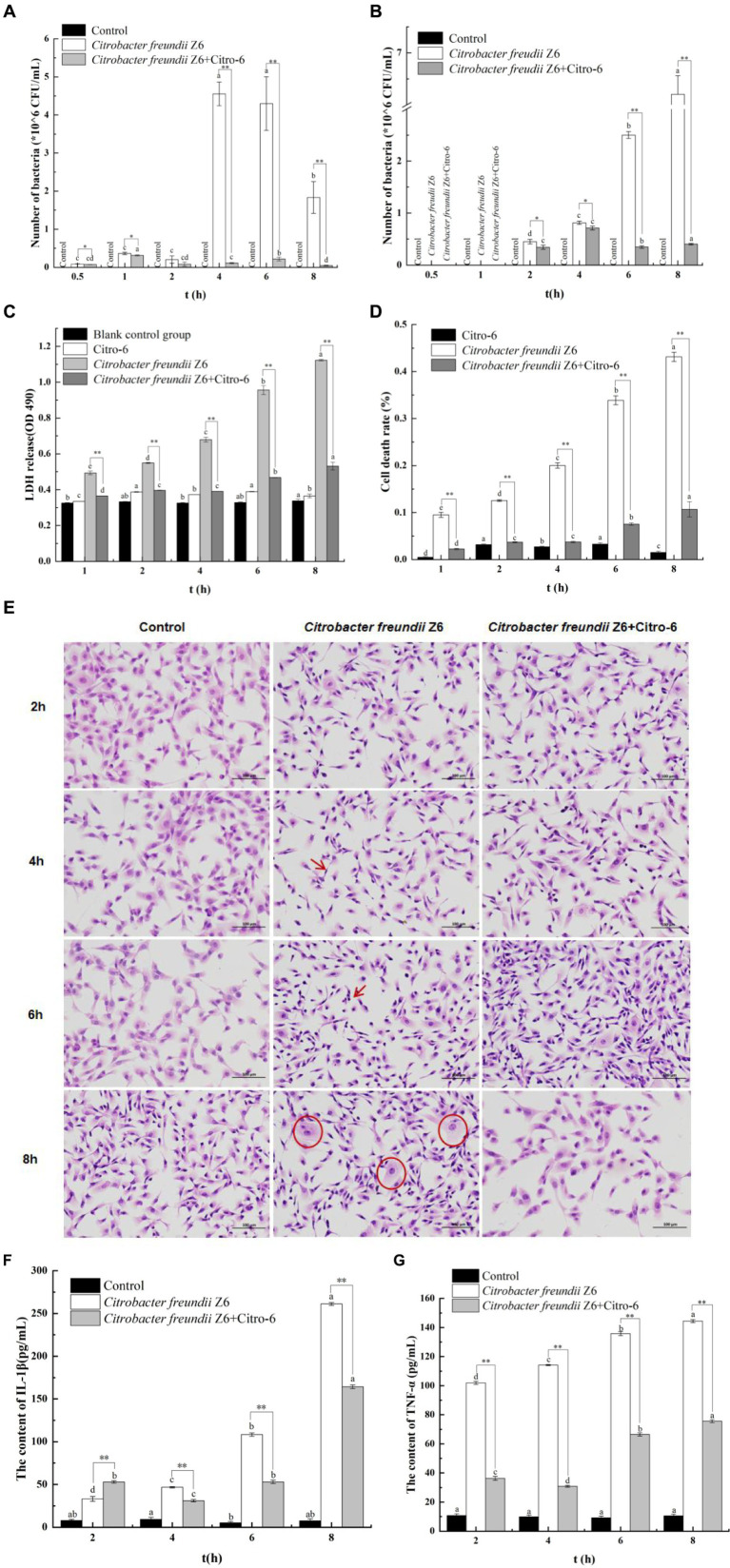
Protective effect of phage Citro-6 on bMECs infected with *C. freundii* Z6 and modulation of pro-inflammatory cytokine release. **(A)** Adhesion of *C. freundii* to bMECs. **(B)** Invasion of *C. freundii* into bMECs. **(C)** Lactate dehydrogenase (LDH) release from bMECs. **(D)** Cell death rate of bMECs. **(E)** Morphological observation of bMECs under different treatments (control, Z6 alone, Z6 + phage Citro-6). **(F)** Concentration of IL-1β in the supernatant of bMECs. **(G)** Concentration of TNF-*α* in the supernatant of bMECs. Data are presented as mean ± SD (*n* = 3). Statistical significance: *p < 0.05, **p < 0.01, compared to the Z6-infected group or as indicated. Different lowercase letters indicate statistically significant differences between groups (*p* < 0.05), while the same letters indicate no significant difference.

Pathogenic bacterium Z6 began adhering to cells at 0.5 h, with invasion detected at 2 h, reaching approximately 4.5 × 10^5^ CFU/mL. Over time, the number of invading bacteria gradually increased, peaking at 6.2 × 10^6^ CFU/mL by 8 h. In contrast, the phage Citro-6-treated group exhibited significantly lower bacterial adhesion and invasion at all time points compared to the positive control group (Figures 5A,B). This indicates that phage Citro-6 rapidly acts on adhered bacteria, inhibiting their early colonization. Simultaneously, it continuously lyses extracellular bacteria, effectively preventing subsequent invasion processes and reducing intracellular infection.

In cytotoxicity assays, the LDH release in the blank control group consistently remained at a low level (OD_490_ ≈ 0.32), with near-zero cell mortality, indicating no significant cell membrane disruption under normal experimental conditions and routine culture practices. In contrast, LDH release in the Z6-infected group increased significantly over time. Elevated LDH release began early in infection (1–2 h), showing a significant difference compared to the blank control group. Between 4–8 h, cell membrane damage intensified further, causing LDH release to surge sharply. At 8 h, OD₄₉₀ reached 1.12, indicating that Z6 infection induces severe membrane damage in bMECs, with cytotoxicity significantly increasing over time. Under identical infection conditions, LDH release in the Citro-6-treated group was significantly suppressed at all time points, consistently maintaining levels close to the blank control group. At 8 h, cell mortality was reduced by approximately 75.2% compared to the Z6-infected group (Figures 5C,D). This demonstrates that phage Citro-6 exhibits no cytotoxicity while effectively lysing Z6, thereby blocking the cytotoxic process induced by this pathogen and protecting host cells.

Morphological observations of bMECs via HE staining provided a visual assessment of cellular structural changes across different treatment groups at various time points (Figure 5E). Cells in the blank control group maintained normal morphology at all time points, exhibiting intact, typical epithelial spindle or polygonal shapes with uniform cytoplasm. The nuclei exhibit fine staining, arranged loosely yet orderly, without shrinkage or rupture, presenting the morphology of normal healthy cells. Cells in the Z6-infected group largely retained their overall morphology at 2 h, with only a very small number exhibiting mild cytoplasmic condensation. No significant early damage was observed, indicating the bacteria were in the colonization phase. Starting at 4 h, cells showed marked shrinkage, nuclear condensation, and morphological abnormalities, with damage progressively worsening over time. By 8 h, a large number of cells had rounded up, forming distinct foci of necrosis and aggregation, with severe disruption of cell integrity. In contrast, cells treated with phage Citro-6 maintained good morphology at all time points, showing no significant shrinkage or condensation. Cell junctions remained intact, with density and alignment comparable to the blank control group, exhibiting only minor abnormalities. This indicates that phage Citro-6 significantly alleviates damage caused by *C. freundii* Z6 to bMECs, demonstrating excellent cell protective effects.

### 3.7 Modulation of inflammatory response

Based on ELISA results, the effects of *C. freundii* Z6 infection on inflammatory cytokine release in the bMECs, as well as the potential anti-inflammatory activity of phage Citro-6, were evaluated (Figures 5F,G). In the blank control group, the concentrations of IL-1β and TNF-*α* remained consistently low throughout the experimental period, ranging from 5 to 10 pg/mL and approximately 10 pg/mL, respectively, indicating the absence of significant inflammatory activation under normal culture conditions. In contrast, cells infected with *C. freundii* Z6 exhibited a sustained inflammatory response, with both cytokine concentrations increasing progressively over time. At 8 h post-infection, IL-1β and TNF-α reached 261.1 pg/mL and 144.4 pg/mL, respectively, representing approximately 35-fold and 14-fold increases compared to the blank control group at the same time point. These results suggest that Z6 infection strongly activates pro-inflammatory signaling pathways in bMECs, leading to substantial release of key inflammatory cytokines. In the Citro-6-treated group, however, the levels of IL-1β and TNF-α were significantly lower and increased at a markedly slower rate. At 8 h, IL-1β concentration was 164.3 pg/mL and TNF-α was 75.6 pg/mL, corresponding to reductions of approximately 37.1 and 47.6%, respectively, compared to the infected group. These findings indicate that phage Citro-6 intervention attenuated the progression of the inflammatory response induced by *C. freundii* Z6.

## 4 Discussion

In the present study, we isolated and characterized a novel lytic phage, Citro-6, specifically targeting *C. freundii* Z6, a strain isolated from clinical bovine mastitis milk samples. To our knowledge, there are few reports describing the biological and genomic features of a phage with lytic activity against *C. freundii* of mastitis origin, as well as its potential to mitigate bacterial adhesion to and invasion of bMECs and to attenuate the associated inflammatory response. Our findings contribute to the expanding body of evidence supporting phage therapy as a viable alternative or adjunct to conventional antibiotics for the control of mastitis caused by Gram-negative pathogens.

Morphological analysis revealed that phage Citro-6 possesses an icosahedral head approximately 54 nm in diameter and a long, slender, non-contractile tail approximately 90 nm in length, consistent with its classification within the family *Siphoviridae* of the order Caudovirales (Krupovic et al., 2016). Members of this family are typically characterized by relatively narrow host ranges, often restricted to specific strains within a bacterial species (Hyman and Abedon, 2010). However, host range determination revealed that phage Citro-6 exhibited lytic activity against 10 of the 12 tested *Citrobacter* strains (83.3%), encompassing diverse species including *C. rodentium*, *C. amalonaticus*, *C. braakii*, *C. youngae*, *C. sedlakii*, *C. koseri* and *C. freundii*. This broad intrageneric lytic spectrum is a notable feature of Citro-6, distinguishing it from many other siphoviruses and enhancing its therapeutic potential against mastitis caused by various *Citrobacter* species. The ability to lyse multiple species within the genus *Citrobacter* is particularly advantageous in the context of bovine mastitis, where etiological diagnosis at the species level may not always be immediately available, and where mixed infections involving different *Citrobacter* species can occur. The broad host range of Citro-6 may be attributed to its recognition of conserved receptor structures on the surface of *Citrobacter* cells, such as lipopolysaccharide or outer membrane proteins, which are shared across multiple species within the genus. This feature aligns with observations from other broadly lytic phages targeting Enterobacteriaceae, where tail fiber proteins capable of recognizing conserved bacterial surface structures mediate cross-species infectivity (Martinez-Soto et al., 2021). The broad intrageneric host range of Citro-6 suggests that, as a monophage preparation, it could potentially address infections caused by a diverse array of *Citrobacter* species, reducing the need for complex phage cocktails in some clinical scenarios. Nevertheless, the development of phage cocktails incorporating Citro-6 together with complementary phages could further broaden coverage and mitigate the emergence of phage-resistant mutants, representing a prudent strategy for therapeutic application (Lauman and Dennis, 2021).

The stability of phages under conditions relevant to the bovine mammary gland is a critical determinant of their therapeutic potential. Phage Citro-6 exhibited remarkable stability across a wide range of temperatures (up to 45 °C) and pH values (5.0 to 9.0), retaining high titers after 1 h of incubation. These characteristics are advantageous for intramammary application, given that the normal physiological temperature of the bovine udder is approximately 37 °C and the pH of milk from healthy quarters ranges from 6.4 to 6.6 (Liu and Chen, 2005). Although a significant reduction in titer was observed at temperatures exceeding 55 °C and under strong acidic (pH ≤ 4.0) or alkaline (pH ≥ 10.0) conditions, such extreme environments are unlikely to be encountered within the mammary gland or during typical storage and handling of phage preparations. The thermal and pH tolerance profile of Citro-6 is comparable to that of previously reported phages active against mastitis pathogens, such as *S. chromogenes* phage PT1-1, *S. gallinarumand* phage PT1-4 and *S. aureus* phages JDYN and JDF86, which also retained activity under similar ranges (Li et al., 2024b; Guo et al., 2024). Furthermore, the stability of Citro-6 under the tested conditions suggests that it could withstand the physicochemical stresses encountered during formulation, storage, and administration, supporting its further development as a therapeutic agent.

Genomic analysis of phage Citro-6 provided critical safety assurances necessary for its potential *in vivo* application. The genome was devoid of genes encoding integrases, repressors, or other components associated with lysogeny, confirming the strictly lytic nature of the phage and mitigating the risk of lysogenic conversion or transduction of virulence factors (Amarillas et al., 2021). Importantly, no homologs of bacterial virulence genes, antibiotic resistance genes, or known toxin-encoding sequences were identified in the Citro-6 genome, aligning with the safety criteria established for therapeutic phage selection (Abd-Allah et al., 2021).

The *in vitro* efficacy of phage Citro-6 was demonstrated through bacterial adhesion and invasion assays using bMECs, which constitute the first line of defense against intramammary infections (Tartaglia et al., 2018). Treatment with Citro-6 significantly reduced the number of intracellular *C. freundii* Z6 recovered from infected bMECs, indicating that the phage can effectively target bacteria that have invaded host cells. This finding is particularly relevant given the ability of coliform bacteria, including *Citrobacter* species, to persist within mammary epithelial cells, potentially evading antibiotic action and serving as reservoirs for recurrent infections (Au et al., 2021). Phages are unable to penetrate eukaryotic cells; instead, they act primarily by rapidly eliminating adherent bacteria, thereby inhibiting early colonization, while continuously lysing extracellular bacteria to effectively prevent subsequent invasion and reduce intracellular infection. This mechanism limits the therapeutic efficacy of phages against infections in which bacteria persist within host cells. However, because phages specifically target prokaryotic cells, they do not affect human or animal cells, representing a key safety advantage (Palma and Qi, 2024). Our results are consistent with those of Guo et al. (2024), who demonstrated that a phage cocktail significantly reduced the internalization of *S. aureus* into MAC-T cells, and with Wang et al. (2021), who reported similar findings for a phage targeting *Pseudomonas aeruginosa*. The observed reduction in intracellular bacterial load likely contributes to the attenuation of the host inflammatory response.

Infection of bMECs with *C. freundii* Z6 resulted in a marked increase in the release of the pro-inflammatory cytokines IL-1β and TNF-*α*, reflecting the activation of innate immune pathways in response to bacterial challenge. Elevated levels of these cytokines have been implicated in the pathophysiology of mastitis, contributing to tissue damage and clinical manifestations (Bannerman et al., 2004). Treatment with phage Citro-6 significantly attenuated the Z6-induced cytokine release, with reductions of approximately 37% for IL-1β and 48% for TNF-α at 8 h post-infection compared with the infected untreated group. This anti-inflammatory effect is likely attributable, at least in part, to the reduction in bacterial burden, as decreased bacterial numbers would result in lower pathogen-associated molecular pattern (PAMP) exposure and subsequent dampening of Toll-like receptor (TLR) signaling (Bhattarai et al., 2018).

Despite the promising findings presented herein, several limitations of this study should be acknowledged. First, although phage Citro-6 demonstrated a broad intrageneric lytic spectrum, lysing 83.3% of tested *Citrobacter* strains encompassing multiple species, all experiments were conducted *in vitro* using a bMEC line. While this system provides a controlled and reproducible platform for evaluating phage efficacy, it cannot fully recapitulate the complex physiological and immunological environment of the lactating bovine udder (Titze and Krömker, 2020). The interactions between phage, pathogen, and host are likely to be influenced by factors present in milk, such as casein micelles, fat globules, and immune cells, which may affect phage stability, diffusion, and antibacterial activity (Gill et al., 2006). Therefore, the efficacy of phage Citro-6 observed *in vitro* requires validation in appropriate *in vivo* models. Second, despite its broad activity against diverse *Citrobacter* species, Citro-6 did not lyse all strains tested (2 of 12 strains were resistant), highlighting the inherent variability in phage susceptibility even within a single bacterial genus. This observation underscores the importance of accurate etiological diagnosis prior to phage intervention and supports the rationale for developing phage cocktails to achieve comprehensive coverage against the heterogeneous population of *Citrobacter* strains that may be encountered in cases of bovine mastitis. Third, the potential for emergence of phage-resistant mutants, although not investigated in the present study, represents a common challenge in phage therapy that must be addressed (Li et al., 2025). The broad host range of Citro-6 may delay but not entirely prevent the development of resistance, necessitating proactive strategies such as cocktail formulation or rotation. Fourth, formulation and delivery challenges specific to intramammary administration remain to be resolved; these include ensuring adequate phage distribution within the mammary gland, maintaining stability in the milk environment, and achieving sufficient residence time to exert therapeutic effects (Saleem et al., 2024). Finally, the absence of *in vivo* toxicity and pharmacokinetic data precludes definitive conclusions regarding the safety and efficacy of Citro-6 for clinical application.

Future research should prioritize the evaluation of phage Citro-6 in a murine mastitis model, which has been extensively used to assess the therapeutic potential of phages against *S. aureus*, *E. faecium*, and *K. pneumoniae* (Brouillette et al., 2023; Ji et al., 2023; Liang et al., 2023). Such studies would provide critical insights into the *in vivo* antibacterial activity, pharmacokinetics, and immunomodulatory effects of Citro-6, as well as its ability to ameliorate mastitis pathology. Should these results prove favorable, subsequent trials in dairy cows would be warranted to establish efficacy under field conditions. Given the narrow host range of Citro-6, the development of a phage cocktail incorporating multiple phages with complementary lytic spectra against pathogenic strains represents a logical next step. Cocktails have been shown to reduce the frequency of emergence of phage-resistant mutants and to enhance bactericidal activity compared with monophages (Kelly and Jameson, 2024). Additionally, exploring the potential synergy between phage Citro-6 and conventional antibiotics could yield combination therapies that exploit different mechanisms of action to improve treatment outcomes while reducing antibiotic selective pressure (Nikolich and Filippov, 2020). Such an approach aligns with the broader goal of integrating phage therapy into existing antimicrobial stewardship programs in veterinary medicine.

## 5 Conclusion

In summary, we have isolated and characterized a novel lytic *Siphoviridae* phage, Citro-6, with specific activity against *C. freundii* Z6, a strain associated with bovine mastitis. The phage demonstrated favorable stability under conditions relevant to the bovine mammary gland, a strictly lytic genome devoid of harmful genetic elements, and the ability to significantly reduce bacterial adhesion to and invasion of bMECs while attenuating the associated inflammatory cytokine response. These findings establish phage Citro-6 as a promising candidate for further development as an alternative or adjunct to antibiotic therapy for the control of *C. freundii*-induced mastitis. However, translation of these *in vitro* findings to clinical applications will require rigorous *in vivo* validation, formulation optimization, and the strategic design of phage cocktails to address the inherent diversity and adaptive potential of target pathogens.

## Data Availability

The complete genome sequence of phage Citro-6 has been deposited in the National Microbiology Data Center (NMDC) under accession number NMDC60315064 (https://nmdc.cn/resource/genomics/genome/detail/NMDC60315064). The 16S rRNA gene sequence of the host bacterium Citrobacter freundii Z6 has also been deposited in NMDC under accession number NMDCN000D0QR (https://nmdc.cn/resource/genomics/sequence/detail/NMDCN000D0QR).

## Footnotes

1
https://nmdc.cn/resource/genomics/sequence/detail/NMDCN000D0QR

2
https://phastest.ca/

3
https://card.mcmaster.ca/

4
https://blast.ncbi.nlm.nih.gov

5
https://nmdc.cn/
