# Supplementary material for: Isolation, characterization, and in vitro efficacy of phage Citro-6 against Citrobacter freundii-induced bovine mastitis
Source: Front Microbiol. 2026 Jul 3;17:1879434. doi: 10.3389/fmicb.2026.1879434 (PMC13376097; doi:10.3389/fmicb.2026.1879434)
Supplement: Supplementary file 1 [file Supplementary_file_1.DOCX]

Lysin

MAIKKTIAATITGAALMMASPLIEEIEGVRYKPYKDIAGIWTVCHGITGKDVILGKEYTRRECDALLAKHMKVAADAVDKAVKVDIPISMRAALYSFTFNAGTGAFSKSTMLKKINNGDLYGGCGELWNWTYYRNPKTGKKEKSKGLKNRRAVEYKYCVMDLNK

Holin

MREFINFATYSSGGASFSGAATGQLMLAVLTFIFFVIFGAVGLWLRWRDSKAIREALECGDLKTALKINQ
